# Review of rhythm disturbances in patient after fontan completion: epidemiology, management, and surveillance

**DOI:** 10.3389/fped.2025.1506690

**Published:** 2025-02-12

**Authors:** Kevin Wall, Camden Hebson, Roshan D’Souza, Seshadri Balaji

**Affiliations:** ^1^ Department of Pediatrics, Division of Cardiology, University of Alabama at Birmingham, Birmingham, AL, United States; ^2^ Department of Pediatrics, Division of Cardiology, Seattle Children’s Hospital, Seattle, WA, United States; ^3^ Department of Pediatrics, Division of Cardiology, Oregon Health & Science University, Portland, OR, United States

**Keywords:** fontan, junctional rhythm, tachyarrhythmia, antiarrhythmic drugs, arrhythmia, congenital heart disease, surveillance

## Abstract

**Purpose:**

The Fontan operation is commonly associated with alterations in heart rhythms, both tachycardic and bradycardic. Despite modifications to attempt to mitigate these complications, arrythmias still frequently occur. The purpose of this review is to examine the literature regarding the scope of the problem, therapeutic options, and current recommendations regarding screening and surveillance.

**Recent findings:**

Modifications to the original Fontan procedure, antiarrhythmic medications, and improvements in catheter ablation procedures have improved the management of patients with arrhythmias following Fontan palliation. There is growing interest in the role of junctional rhythm in the role of Fontan dysfunction. While chronotropic incompetence has often been blamed for poor exercise testing, there is evidence that decreased performance may be related to ventricular filling and Fontan hemodynamics.

**Summary:**

Tachyarrhythmias are an important cause of mortality and morbidity after the Fontan operation. Prompt and aggressive management of arrhythmias with the goal of maintaining sinus rhythm is vital. Management strategies such as anti-arrhythmic medications, ablation, anti-tachycardia pacing and Fontan conversion should be seen as complementary and used early to prevent hemodynamic deterioration. Bradyarrythmias likely also contribute to Fontan failure. Pacing is the primary management strategy with evidence supporting use of atrial pacing. However, ventricular pacing seems to often lead to deleterious effects. Current guidelines recommend surveillance with Holter monitor every 2–3 years in adolescents and every 1–2 years in adults. Future directions for research include further assessment of junctional rhythm and its management as well as further identifying patients in which pacing would be beneficial.

## Introduction

The Fontan operation transformed the outlook for children born with a single ventricle. However, it is a fragile circulatory arrangement with a tendency to develop a multitude of problems (1). Specifically, arrhythmias are one of the commonest causes of morbidity and mortality in these patients (1). Despite surgical modifications like the intracardiac lateral tunnel (ILT) and the extracardiac conduit (ECC) Fontan, arrhythmias continue to occur in a significant number of patients (1). Arrhythmias may present in a wide variety of ways ranging from the subtle (vague or mild symptoms of fatigue) to the catastrophic (syncope or rarely, cardiac arrest). Some, with arrhythmias may even be detected only at routine follow up. Yet another important consideration in Fontan patients is their risk for acute thromboembolic events (1–3). Underlying structural problems including poor ventricular function, and valve regurgitation may predispose to arrhythmias. Arrhythmias also can lead to worsening of cardiac ventricular function. One report showed that ∼40% of patients developed ventricular dysfunction after the first onset of an arrhythmia (1, 4, 5). Supraventricular tachycardia (SVT) has also been shown to be associated with a six-fold increase in transplantation and death (1).

Physicians caring for these patients must be aware of these arrhythmias, including their unique presentations after the Fontan, the diagnostic approach one should take, and the treatment options available. Regular surveillance to detect pre-clinical arrhythmias and prompt recognition and management of clinical arrhythmias are keys to achieving this goal. In this review, we aim to review tachyarrhythmias and bradyarrhythmias that arise after Fontan palliation, including a summary of the literature and an up-to-date approach to these often-challenging clinical presentations.

## Tachycardia in patients after fontan completion

In the original, so-called atrio-pulmonary connection (APC) Fontan, the presence of extensive surgical scars in the right atrium (RA) combined with pressure and volume overload of the RA (which lead to stretching of the scars) is thought to predispose to the development of arrhythmias (6–10). While surgical scars are still present after the ILT and the ECC, they are fewer. Also, the ILT leaves most of the RA in the lower pressure pulmonary venous side of the atrium (11, 12). The ECC leaves the entire RA on the low pressure pulmonary venous side (13). On the other hand ventricular tachyarrhythmia (VT) is predominantly seen in patients with ventricular surgical scars and in those with a dominant right ventricle (14, 15).

The development of any tachyarrhythmia is an independent predictor of poor clinical outcome and tachyarrhythmias are associated with Fontan failure, sudden cardiac death) SCD, and mortality (4, 5, 9, 16–20). In one study, the 15-year survival after development arrhythmias was 70% and freedom from Fontan failure was 44% (20). Atrial arrhythmias are associated with a six-fold increase in transplantation and death (21).

**Figure 1:**
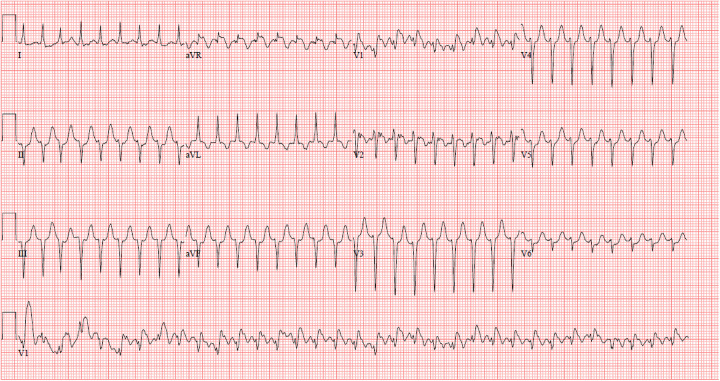
IART in a young adult with intra-cardiac lateral tunnel fontan.

Given the fragile nature of the Fontan circulation, prompt recognition and management of tachyarrhythmias are key to improving patient quality and quantity of life. Some arrhythmias, especially intra atrial reentry tachycardia (IART) may appear innocuous and resemble either sinus or a “junctional” tachycardia on electrocardiogram (22, 23). An example of IART may be seen in Figure 1. The presence of extensive scarring in the atrium can lead to low amplitude and fractionated p waves which may be hard to see (22, 23). A high index of suspicion is important in making a timely diagnosis. In the next sections, we review the various types of tachyarrhythmias.

### Supraventricular tachycardia

The APC type of Fontan is associated with a high incidence of SVT. SVT occurred in the early post-operative period in 10%–30% of patients followed by a steady incidence during follow-up (24–28) Late arrhythmias occurred in up to 80% by >30 days post operative period (median time of 12 years after surgery) (27–29). Early post-operative SVT occurs in 25% patients after the ILT (30) and 14% after the ECC Fontan (29). As for late post-operative SVT, it has been noted in 32% of ILT and 6%–17% of ECC by 1–5 years post-operative (1, 24–28).

Much of what we know about the specific type of SVT's seen in these patients comes from studies describing outcomes after catheter ablation for SVT (21). The most common type of SVT in these patients is IART (∼93%) (21, 31, 32). Patients, however, can also have atrioventricular re-entrant tachycardia (AVRT) and atrioventricular nodal re-entrant tachycardia (AVNRT) which occur in ∼4% (21). As patients age, atrial fibrillation becomes the dominant arrhythmia, and has been noted in −2%–40% (4, 9, 33). However, other mechanisms such as AVRTs utilizing either an accessory pathway or twin atrioventricular nodes, AVNRT, ectopic atrial tachycardias, and junctional tachycardia are described (34).

### Medical management of SVT

Prompt treatment is a must for episodes causing significant hemodynamic compromise. However, even in those who may tolerate the tachyarrhythmia acutely, conversion to sinus rhythm as soon as possible is of utmost importance (22). The importance of atrial kick in maintaining adequate cardiac output in the Fontan circulation cannot be overstated. Therefore, in patients with the Fontan physiology, the goal should be rhythm control rather than rate control (35).

For acute conversion, adenosine can be used for SVT involving the atrioventricular node after evaluating baseline ventricular function with an echocardiogram and electrocardiogram. However, such SVT are less common. Therefore, most patients with IART or atrial fibrillation need cardioversion (22). Most centers use direct current cardioversion. If the patient is stable with an adequate blood pressure, medications can be tried before cardioversion, although it is not considered first line therapy. Options for pharmacologic cardioversion include intravenous (IV) Ibutilide, IV sotalol, IV procainamide, and IV amiodarone (35–37). Due to the risk of development of intracardiac thrombi with untreated atrial arrhythmias >24–48 hour duration, pre-procedural transesophageal echocardiogram (to assess for thrombus), anticoagulation, and conscious sedation is necessary (38, 39).

Chronic therapy options include anti-arrhythmic drugs, catheter ablation, anti-tachycardia pacing and the surgical MAZE procedure (35). A summary of medication options for long term management of IART including dosage, side effects, and monitoring considerations can be found in Table 1. The goal of chronic medical management is maintenance of sinus rhythm. The choice of medication primarily depends upon whether or not there is normal ventricular function. If so, class 1c agents (propafenone or flecainide) or sotalol (class III agent) are typically chosen as first line medication. If not, amiodarone or dofetilide may be considered. Amiodarone may also be employed as a second line agent in patients with normal ventricular function (35).

**Table 1:** Chronic rhythm control in adults with fontan circulation and IART.

Medication	Indication	Dose	Side effects contraindications	Monitoring issues
Flecainide (class IC)	First line in patients with normal ventricular function	Dose adjusted based on serum levels: Initial: 50 mg bid Increase by 50 mg bid at 4-day intervals; maximum dose: 300 mg/day	Black box warning with increased risk of ventricular arrhythmias as well as mortality in patients with ventricular dysfunction or coronary artery disease Proarrhythmic (VT, bradycardia, AV block) QRS prolongation Has potential to convert atrial arrhythmias into 1:1 conduction due to negative chronotropic effects—should initiate concurrent AV node blocking medication	Dosing based on serum levels which need to be intermittently monitored: therapeutic trough concentration is between 0.2 and 1 mcg/ml Follow daily ECG for QRS prolongation while inpatient until therapeutic
Propafenone (class IC)	First line in patients with normal ventricular function	Immediate release: Initial: 150 mg every 8 h; may increase after 3- to 4-day intervals; may increase to 300 mg every 8 h. Extended release: Initial: 225 mg every 12 h; may increase after 5-day intervals; may increase to 425 mg every 12 h	Black box warning with increased risk of ventricular arrhythmias as well as mortality in patients with ventricular dysfunction or coronary artery disease Proarrhythmic (VT, bradycardia, AV block) QRS prolongation Has potential to convert atrial arrhythmias into 1:1 conduction due to negative chronotropic effects—should initiate concurrent AV node blocking medication Central nervous system side effects such as dizziness, nausea, unusual taste, and blurred vision	Check ECG for QRS prolongation daily while inpatient until therapeutic
Sotalol (class III)	First line in patients with normal ventricular function	Initial dose: 80 mg Dosing frequency based on calculated creatinine clearance: > 60 ml/min: bid dosing 40–60 ml/min: daily dosing < 40 ml/min: contraindicated Adjustment: may increase every 2–3 days up to 320 mg, given in 2 or 3 divided doses	Can prolong the QT interval, so check QT interval before administering (contraindicated if > 450 ms) Proarrhythmic (torsade); exercise caution when combining with other QT-prolonging medications (antimicrobial, antiemetic, etc)	Check ECG for QRS prolongation daily while inpatient (adjust dose if QTc > 500 ms) Monitor ECG intermittently once stable
Amiodarone (class III)	First line in patients with ventricular dysfunction Second line if function is normal	200 mg daily	Pulmonary and liver toxicity, corneal microdeposits, photosensitivity, thyroid dysfunction [hypo- or hyperthyroidism, especially in women post Fontan and those with BMI < 21 kg/m2 (35)], and adverse cardiac effects (e.g., bradycardia, torsades de pointes)	Cardiac: baseline testing of ICD threshold if one present Pulmonary: chest radiograph at baseline and yearly for asymptomatic patients; PFT if symptoms develop Thyroid: baseline TSH and FT4 at baseline; serial testing 3–4 months post initiation, then yearly Liver: baseline AST/ALT, repeat 6 months post initiation, then yearly Ophthalmologic: yearly eye exam
Dofetilide (class III)	Second line therapy as alternative to amiodarone in patients with ventricular dysfunction	Dofetilide dosing is based on creatinine clearance and calculated QTc: Initial dose with calculated creatinine clearance: > 60 ml/min: 500 mcg bid 40 –60 ml/min: 250 mcg bid 20 –40 ml/min: 125 mcg bid < 20 ml/min: contraindicated Subsequent doses adjusted if QTc increases > 15% or QTc > 500 ms Starting dose: adjusted dose: 500 mcg bid 250 mcg bid 250 mcg bid 125 mcg bid 123 mcg daily 125 mcg daily	Contraindicated in pregnancy (class C), patients with LQTS, patients on dialysis or with renal disease, or with vomiting or electrolyte derangements Adverse Effects: Proarrhythmic—Torsades de pointes in 1–3% of patients, may induce of worsen ventricular dysrhythmias, possibly inducing PMVT Side Effects: Rash, diarrhea, dizziness, sweating, vomiting, loss of appetite	Follow QTc to monitor for signs of prolongation. FDA recommends admission for initiation with continuous ECG and calculating QTc 2–3 h after doses 2–5 after starting and monitor for minimum of 3 days

IART, intra atrial reentry tachycardia; AV, atrioventricular; ECG, electrocardiogram; VT, ventricular tachycardia; PMVT, polymorphic ventricular tachycardia; QTc, adjusted QT interval; LQTS, long QT syndrome; PFT, pulmonary function testing.

### Catheter ablation

Catheter ablation of SVT in Fontan patients is a highly specialized procedure performed by electrophysiology physicians with training and experience in congenital heart disease (18). These procedures entail prolonged case time, are complex, and require highly specialized equipment including three-dimensional mapping (3D) technology, intracardiac echocardiogram and the use of specialized catheters. With large atrial size, multiple scars and pathways, the APC Fontan patients pose significant challenges during ablation as there may be multiple circuits with the rhythm morphing from one to the other (36, 40). In APC Fontan patients, studies have described an acute success rate varying from 78%–94%, with partial success achieved in 6%–13%. The recurrence rate is high (20%–50%), and multiple ablations are needed in ∼5% of patients (21, 40–42). Complications of ablation include complete atrioventricular block requiring pacemaker placement, thrombus and embolism, rarely fatality (36, 40–42). Despite these risks at 24 months following ablation, there was an improvement in overall arrhythmia score. This is likely postulated due to either ablation of all tachycardia foci or modification of the dominant arrhythmia substrate (21, 42). The acute success rate has been described to be higher in ILT patients (18), however, they also have a high recurrence rate (18). ECC patients require puncture of the Fontan baffle to access the atrial myocardium, without significant consequent risks (42). In the ECC group, the most common site of arrhythmias is the cavo-tricuspid isthmus, with consequently higher success rates for ablation (43). Reports of ablation of AVNRT and AVRT using an accessory pathway or twin AV nodes are confined to small case series (42). Any ablation in a Fontan patient must surmount a few anatomical challenges and therefore must be performed at specialized centers with experienced personnel.

### Summary of SVT management

It is our practice that an in-depth discussion is pursued with the family regarding ablation vs. medical management, emphasizing that these therapies are complementary and not contradictory; most patients will need both. After a detailed conversion with shared decision making, we would proceed with either a class 1C or class III medication for rhythm control. It is our practice to typically admit patients for initiation of medication over the course of 2–3 days to ensure stability. If they tolerate this, we continue therapy as an outpatient with frequent ambulatory monitoring to assess adequacy of treatment. In select patients, catheter ablation may be considered as a first line therapy. When ablation is successful, patients may be monitored without need for initiating anti-arrhythmic therapy. If unsuccessful or only partially successful, we would initiated medications and monitor as above.

### Ventricular tachycardia

Ventricular tachycardias are less frequent in the Fontan population (3%–10%) and non-sustained ventricular arrhythmias are typically discovered during surveillance (6, 13). However, the long-term implications of this arrhythmia, especially in relation to risk for sudden death, is unclear. This is an important consideration given that reports show that the mode of death is sudden (and presumably arrhythmic) in −3%–12% of Fontan patients (2, 24, 25, 33).

VT can be difficult to manage in Fontan patients. While beta blockers may provide some protective effect, they cannot be relied upon to be the mainstay of treatment in patients who have VT associated with major symptoms such as syncope or near-syncope (13, 16, 44). ICD placement may need to be considered. Given the anatomical constraints imposed by the Fontan circulation, ICD's are hard to place (41, 42, 45–47). The subcutaneous ICD may be an excellent option in many patients (42). Major anti-arrhythmic drugs such as sotalol and amiodarone should be considered in patients with VT.

### Anti-arrhythmic drugs

Vaughan-Williams class Ic/III (Sodium and Potassium channel blockade) rhythm-control agents are initiated in the hospital with electrocardiogram monitoring. Class I agents include flecainide and propafenone and class III agents commonly used are Amiodarone, Sotalol and Dofetilide (36). Sotalol and Dofetilide can cause prolongation of the QT interval and torsades as a lethal proarrhythmia (35, 48, 49). Hence, careful monitoring of the QT at the time of initiation of these two drugs is critical to long term use (49). Amiodarone, even though is most effective drug in the long-term, is disadvantageous for multiple reasons, especially its association with multiple side effects when used long term (35, 50). Therefore, it is important to think of an “exit-strategy” (such as ablation, Fontan conversion surgery, and transplantation) out of chronic amiodarone use. If possible long-term use should be avoided.

Pharmacologic therapy is associated with a >90% arrhythmia recurrence rate within 3 years (36). Complete control of arrhythmia was seen in 63% whereas 35% of partial benefit was seen with medical therapy alone (50). Discontinuation due to toxicity is common accounting for 42% of AAD (50). In one cohort on Amiodarone, 30% developed thyrotoxicosis and 14% hypothyroidism (35, 50).

Class 1 c agents must be used with caution for two main reasons. Firstly, they can cause QRS duration prolongation and ventricular arrhythmias (35). Secondly, they can slow down the rate of atrial arrhythmias, which can be conducted at a higher ratio to the ventricle. For instance, a stable patient with a IART rate of 300, and 2:1 conduction giving a ventricular rate of 150 may become unstable if their IART rate is reduced (for example to 200) with consequent 1:1 atrioventricular (AV) nodal conduction. Hence class1c agents should always be combined with high doses of AV node blocking agents when used in this patient population (35).

### Fontan conversion

The Fontan conversion operation is an option for patients with an APC faced with intractable arrhythmias. This includes resection of the enlarged RA, atrial septectomy, right-sided or bi-atrial maze cryoablation, extracardiac conduit placement (inferior vena cava to pulmonary artery), bidirectional Glenn, and, if needed, placement of an epicardial pacemaker (8). This surgery has a procedural mortality risk of 0.9%–3% in the perioperative period and a further late mortality 3–5.4% (36, 51). However, patients have been shown to have a freedom from atrial tachycardia of 77% at 10 years and freedom from cardiac death/transplant of 90% at 5 years, 84% at 10 years, and 66% at 15 years (52). High-risk characteristics for the operation at the conversion include right dominant ventricle, cardiopulmonary bypass (CPB) >240 min, ascites, protein losing enteropathy (PLE) and those having bi-atrial MAZE operation (52).

The 2014 HRS/PACE guidelines recommended a modified right atrial MAZE procedure in all Fontan conversion surgeries, including those without prior atrial arrhythmias (35). On the other hand, a left atrial/biatrial Cox MAZE III procedure is chiefly used in those with a known left atrial arrhythmia or atrial fibrillation (AF) (35, 51). Patients who are felt to be too high risk for a Fontan conversion operation, especially those with severe ventricular dysfunction or PLE, should be referred for transplantation.

### Sudden death

Arrhythmias as a cause of SCD has been noted to have an incidence of 3%–12% and occur during late follow-up (6, 13, 16, 53). Risk factors for sudden death include atrioventricular valve replacement at time of Fontan operation and a Fontan pressure >20 mmHg (6). Conversely, the presence of pre-operative sinus rhythm has been shown to be protective (17). Risk stratification to predict SCD is still in its infancy and further studies are needed to identify which patients would benefit from implantable cardioverter defibrillator (ICD) placement.

### Antitachycardia pacing & ICD

Antitachycardia pacing can be used to overdrive pace the patient out of SVT, specifically IART (3). The enthusiasm for antitachycardia pacing has waned after the introduction of catheter ablation. However, it can still have a role in select patients with recalcitrant arrhythmias (3). Patients with resuscitated cardiac arrest or hemodynamically unstable ventricular arrhythmia are usually treated with an ICD. Despite SCD being the cause of death in −20%–25% of adult patients with a Fontan, the lack of adequate risk prediction methods makes it difficult to place a prophylactic ICD in these patients.

### Conclusions

Tachyarrhythmias are an important cause of mortality and morbidity after the Fontan operation. While the change from APC to ILT/ECC has reduced the incidence and hemodynamic deterioration associated with atrial arrhythmias, they have not eliminated the problem. An important key to early detection is a high index of suspicion. Prompt and aggressive management of arrhythmias with the goal of maintaining sinus rhythm is vital. Management strategies such as anti-arrhythmic medications, ablation, anti-tachycardia pacing and Fontan conversion should be seen as complementary and used early to prevent hemodynamic deterioration.

## Bradycardia in patients after the fontan procedure

Bradyarrhythmias present a challenge in patients after completion of the Fontan procedure with clinical complications that can be highly consequential. While the association between tachyarrhythmias and poor outcomes is well-established in the Fontan population, bradyarrhythmias may also have serious clinical consequences, as even small alterations in the hemodynamics of these patients can lead to adverse outcomes including Fontan failure, Fontan associated liver disease, and even mortality (54, 55). While tachyarrhythmias are often easily recognized (due to the severity of their symptoms), bradyarrhythmias can be insidious, with prolonged periods of being asymptomatic prior to discovery (33, 56). A study by Carins et al. from the Australia-New Zealand Fontan registry demonstrated a high incidence of Fontan failure after onset of bradyarrhythmias including sinus node dysfunction (SND) and AV node disease causing heart block (33). In this section, we will review the existing literature on bradyarrhythmias in patients with Fontan physiology.

### Sinus node dysfunction

SND is the most commonly encountered bradyarrhythmia in patients after Fontan completion with an estimated incidence between 9%–60% (56–62). However, this underestimates the issue, as patients with sinus node dysfunction can be asymptomatic (33). SND can be defined as an average resting heart rate greater than two standard deviations below the mean, predominant junctional rhythm, and/or sinus pauses of 3 or more seconds (63). SND typically begins subtly with progressively decreasing periods of sinus rhythm and greater periods spent in bradycardia and junctional rhythm. While sinus bradycardia and pauses may cause symptoms such as fatigue, exertional shortness of breath or dizziness/syncope, many patients “adapt” to their chronic disability and may not endorse any symptoms (64).

The APC Fontan is known to have a high incidence of SND, up to 50% at 10–15 years (12). The high frequency is attributed to the extensive atrial tissue manipulation required during surgery, which likely leads to trauma to the sinus node or compromise of the arterial supply (58, 63). Others have proposed theories to explain why the incidence of sinus node dysfunction increases with including abnormal right-sided hemodynamics, atrioventricular valve regurgitation leading to atrial distention, and multiple suture lines near or around the sinus node (63). Alternative surgical techniques including the ILT and ECC have sought, in part, to mitigate against these risk factors (63, 65–67).

With the ILT, the placement of an intra-atrial baffle leads to less of the atrial wall exposed to higher pressure and thus arrhythmia-generating distension (68). The downside is that there is suturing close to the sinus node during the baffling process. In contrast, the ECC eliminates the need for dissection and suture lines near the SA node as well as protecting against atrial distention (68). However, the need for harvesting an atrial cuff to allow for inferior anastomosis may lead to a higher-than-expected incidence of arrhythmias (18, 69). Despite these alterations in surgical technique, SND occurs in both the immediate post-operative period as well as late term follow-up.

### Early SND

The incidence of SND in the immediate post-operative (Fontan) period is variably estimated between 2% and 25% (56, 59, 63, 68, 70, 71). While a majority of patients do not require pacing prior to discharge, there is evidence that the presence of SND within the first several days post-operatively may predict the development of both late onset sinus node dysfunction and tachyarrhythmias (62, 65, 70, 72). Studies have compared the rates of early SND after each approach to Fontan completion, with conflicting results. Three groups found a higher incidence in the ILT compared to ECC patients (60, 73, 74), with each of these being retrospective single center studies. Conversely, several studies have demonstrated no significant difference between the techniques or even a higher incidence in the ECC patients (70, 71, 75–78). These were also retrospective studies, but include several multicenter and multi-national cohorts. A metanalysis from Li et al. in 2017 included each of the afore-mentioned studies and examined the existing data from these studies (18). The conclusion was that while odds of SND was lower in the ECC group, this did not reach statistical significance (*p* = 0.06) (18).

### Late SND

Late onset SND has a reported incidence between 15% and 44% (56, 57, 79). There is evidence that the presence of early SND predicts the development of late SND (63), and several studies have demonstrated that SND becomes more prevalent over time (63, 70, 80). Any of the estimate of the incidence of SND likely underestimates the true frequency, given to the presence of asymptomatic patients.

Late SND associated with an APC Fontan has been reported in 10%–12% of patients (80). As with early onset SND, multiple studies have compared the incidence of late SND associated with the ILT vs. ECC procedure. Dilawar et al. reported a higher frequency of SND after ECC over a seven-year period in 4/19 ILT patients (21%) vs. 10/17 ECC patients (59%; *p* = 0.04) (58). However, this was a single-center retrospective study with longer mean follow-up in the ECC compared to the ILT group. In contrast, Nürnberg et al. found that late SND was higher in patients after ILT, with 50% of patients following ILT being in a non-sinus rhythm at follow up compared to 14% for ECC patients (60). However, in this study, the median follow up post-ECC was 4.4 years compared to 7.9 years for ILT (60). Overall, a majority of studies including multi-center studies and the meta-analysis from Li et al. have demonstrated no statistical difference in late SND between the two surgical approaches (18, 23, 68, 71, 75, 77, 78).

### Sinus node dysfunction or secondary limitation—insights and the role of exercise testing

Multiple studies have shown that Fontan patients have an abnormal heart rate response to exercise. However, it is unclear if this is a primary rhythm problem or a secondary adaptive response. Powell et al. showed that in teenage Fontan patients, chronotropic limitation to exercise, as defined by chronotropic index [CI = actual peak heart rate (HR)—resting HR/expected peak HR for age—resting HR], was prevalent, with the mean CI being 0.72 as compared to a typical definition of chronotropic incompetence, which is a CI <0.8 (81). The CI, however, is an incomplete assessment of chronotropic function, as it only uses the peak HR (and not slope of HR change) and does not consider secondary reasons for peak HR truncation.

An alternative proposal is that in select Fontan patients, limitation in HR, especially during exercise, is a secondary and even adaptive phenomenon. Support for this comes from Claessen et al, who compared ten patients after Fontan palliation (mean age 20 years, 60% male) to healthy controls utilizing MRI and simultaneous invasive arterial pressure recording during exercise (82). Exercise associated cardiac index, stroke volume, and HR relative to peak oxygen consumption (VO2) were determined and compared amongst the groups. As expected, heart rate reserve (peak HR—resting HR) was lower in Fontan patients compared to controls (71+/−21 vs. 92+/−15 bpm; *p* = 0.01) (82). However, increase in HR relative to workload and peak VO2 were actually higher in the Fontan patients. Furthermore, the Fontan patients had reduced augmentation of stroke volume for any given change in VO2 (82). Finally, the Fontan patients had a pronounced plateau in cardiac output at a lower HR than controls (82). The authors concluded that HR response to exercise was actually appropriate relative to exercise intensity (implying lack of chronotropic incompetence). However, premature reduction in ventricular filling and thus stroke volume led to leveling off of cardiac output during exercise, thus making peak HR secondarily truncated (82). Hedlund et al. reported similar outcomes from their prospective cohort study. When comparing 27 teenage Fontan patients to healthy controls, the Fontan cohort's mean HR for any given workload was actually higher than the controls (83). Furthermore, oxygen pulse, a surrogate marker for stroke volume, was reduced at maximal effort in the Fontan patients comparatively (83). The authors postulated that this was related to reduced ability to augment stroke volume with more intensive exercise and thus the reduced HR was appropriate for the degree of stroke volume (83). Hebert et al. similarly concluded that low stroke volume index was the most important limiting factor for exercise capacity in Fontan patients (84). But how might this heart rate modulation actually occur? A proposal is that this occurs via the Bainbridge and “Reverse” Bainbridge reflexes. These reflexes are described within the anesthesia literature (85), given the HR changes that are routinely observed with changes in peri-procedural fluid status. Via stretch fibers near the vena cava and within the right atrium, excessive cardiac filling and overdistension is prevented via reflex tachycardia (in turn via inhibition of vagal outflow and enhancement of sympathetic stimulus to the sinus node). The “Reverse” Bainbridge is the opposite—reduced venous return leads to deactivation of sinus node stimulation and in the midst of exercise, a negative stimulus for further HR elevation (85). In patients with worse Fontan hemodynamics (elevated central venous pressure and ventricular filling pressure), the already preload-deficient systemic ventricle (given the lack of a subpulmonary ventricle to augment pulmonary blood flow and thus pulmonary venous return to the heart) would be even more lacking in preload, thus leading to a lack of further stimulus to increase HR further during exercise (85).

**Figure 2:**
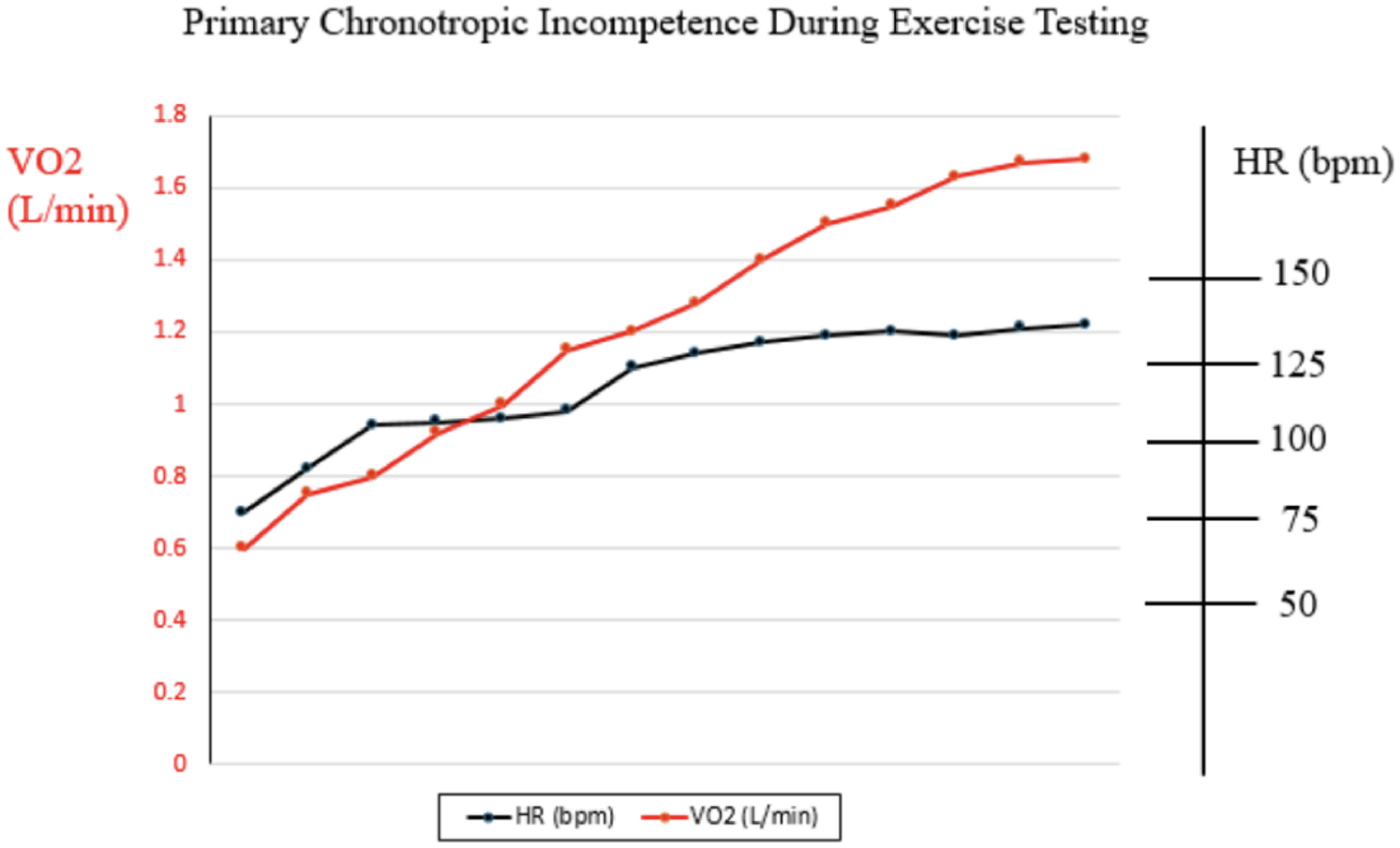
Example of relationship between heart rate and oxygen consumption during cardiopulmonary exercise test in patient with primary chronotropic incompetence.

**Figure 3:**
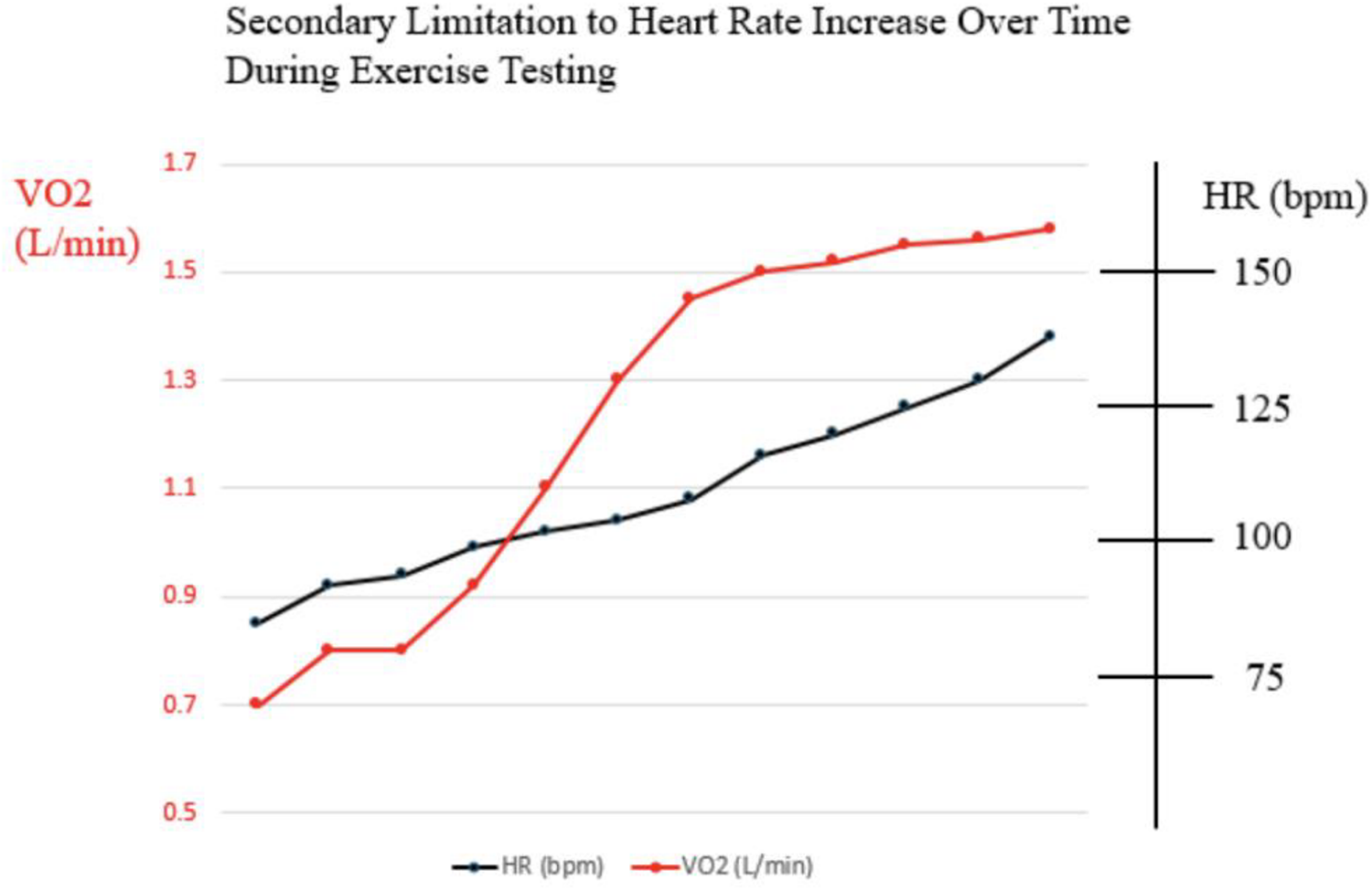
Example of relationship between heart rate and oxygen consumption during cardiopulmonary exercise test in patient with secondary limitation to heart rate such as poor fontan hemodynamics.

Exercise testing is an excellent modality to utilize to help decipher whether HR limitation during exercise is primary or secondary to poor Fontan hemodynamics. In patients with primary sinus node dysfunction, heart rate during exercise would be expected to lag in general, and in comparison with oxygen consumption (VO2). Heart rate might even plateau prior to peak VO2 doing so (Figure 2). On the other hand, limitation in HR secondary to poor Fontan hemodynamics would be expected to show appropriate HR acceleration for any given increase in peak VO2, but abrupt truncation of HR at peak exercise rather than any plateau (Figure 3). We believe complete evaluation of Fontan status, including exercise testing, has a role in deducing the etiology of HR limitation in a given patient and should be considered prior to pacemaker placement.

### Junctional rhythm

**Figure 4:**
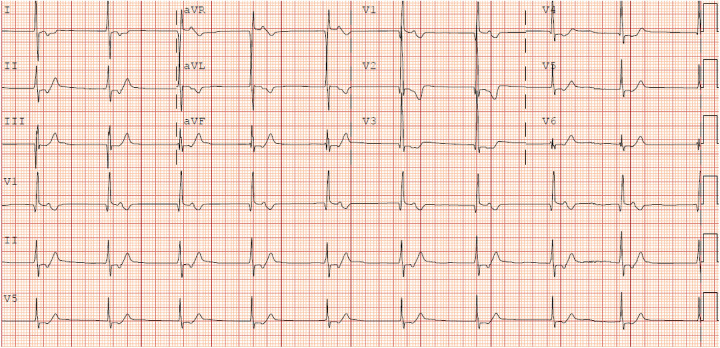
12 lead of junctional rhythm in a fontan patient.

SND manifests as sinus bradycardia, pauses, and junctional rhythm (JR). JR can lead to asynchrony and loss of atrial kick, with further decrease in cardiac output (86). This is particularly important in the Fontan circulation given the tenuous hemodynamics present. JR is also often characterized by an insidious onset. Even more problematically, patients with JR may have a relatively normal heart rate which can further delay the recognition of this arrhythmia (33). An example of an ECG with JR in a Fontan patient is presented in Figure 4. The frequency of JR after Fontan also tends to increase with time, with estimated incidence between 15% and 46% (56, 62, 65, 77, 80, 87). Furthermore, Januszewska et al. compared the rates of JR between the ILT and ECC. In this retrospective cohort study, ILT demonstrated significantly higher rates of JR early postoperatively (*p* < 0.001), during hospitalization (*p* = 0.004), and at discharge (*p* < 0.001) (87).

Evidence for the deleterious effects of JR in the Fontan population comes from several studies. Ferrari et al. reported cardiac magnetic resonance-derived flow patterns showing retrograde flow in the entire Fontan system in a patient with JR (88). This flow reversal increases the pulmonary artery pressure, which in turn increases the hepatic and splanchnic venous pressures, which is particularly deleterious in the Fontan circulation (89). These studies suggest JR chronically decreases cardiac output and further elevates central venous pressure, known risk factors for developing Fontan failure (54). However, it is worth noting that each of these studies are either case reports or small series and the long-term impact of JR remains to be studied.

The optimal management of JR is unclear. In a recent survey of congenital cardiologists, 80% would not implant an atrial pacemaker in an asymptomatic patient with JR (90). On the other hand, more than 83% indicated that they would pursue pacemaker placement if the asymptomatic patient was already undergoing a sternotomy for another reason (90). This suggests that congenital cardiologists feel the risk of pacemaker placement is not worth the uncertain benefits to a patient with JR, although they also understand intuitively the possible benefit of treatment (90). There was general agreement that Fontan patients with symptomatic JR including evidence of Fontan failure should receive pacing, as 90% indicated they would support placement of a device (90).

### Is pacing the solution for the bradycardic fontan?

The direct solution to managing significant bradycardia is pacing. The incidence of permanent pacemaker placement in the Fontan populations is 8%–13% (91–93). Additional reasons for placement following Fontan completion include anti-tachycardia management, ventricular tachycardia, and cardiac resynchronization (92).

There is evidence that permanent pacing is associated with clinical improvement and is recommended in symptomatic patients and those with exercise intolerance, or if there are hemodynamic derangements worsened by bradycardia (1, 35, 91). Cohen et al. reported two patients in whom atrial pacing (AP) resolved symptoms of PLE (94). In these two patients, symptoms remained despite aggressive diuresis and creation of a fenestration in the Fontan circuit (94). In contrast, there was complete resolution of PLE within 6 weeks of AP (1). Heinemann et al. also reported improvement in PLE symptoms after placement of dual-chamber pacemakers in their case series (95). A separate report also described resolution of plastic bronchitis with AP (96). While this total experience is small, AP seems to offer Fontan patients clinical improvement when appropriately utilized.

Pacing has also been shown, via restoration of AV synchrony, to eliminate the reversal of fenestration flow, lower left atrial pressure, and improve cardiac output and clinical status in Fontan patients previously in junctional rhythm or undergoing ventricular pacing (89). In a recent study, Alnoor et al. reported 7 patients with Fontan physiology and JR undergoing cardiac catheterization (97). Hemodynamic measurements were performed in JR and during AP∼10 beats faster than the JR rate. JR was associated with lower cardiac output and elevated central venous pressure, which improved with AP (97). AP increased CO (by∼23%), from 2.7 ± 0.8 (in JR) to 3.5 ± 1 L/min/m2 (97). AP also decreased left atrial pressure (from 8.8 ± 2.6 to 5.5 ± 2.9 mmHg) and increased the pulmonary blood flow (97).

Not all studies, however, have shown a benefit to pacing. Ventricular pacing, in particular, seems to be problematic. A large Pediatric Heart Network study showed that ventricularly paced Fontan patients had worse clinical outcomes and decreased ventricular function compared to those who were not paced (93). Multiple studies have concluded that asynchronous ventricular pacing is particularly deleterious (91, 92, 98–100). Barber et al. found that systemic blood flow was significantly lower with VOO vs. AOO or DOO pacing and theorized that pacing from the ventricle leads to similar physiology to junctional rhythm, where the loss of synchrony leads to a loss of atrial kick (and thus decreased preload to the single ventricle and further reduction in stroke volume) (91). Poh et al. examined the Australian and New Zealand Fontan registry and demonstrated that amongst all paced patients, those with QRS interval >130 ms on electrocardiogram were at greater risk of death and transplantation (92). Furthermore, they found that patients who had ventricular pacing >50% of the time were at higher risk of death, transplantation, and moderate to severe systemic ventricular dysfunction at latest follow-up (92). Poh also showed that atrial pacing was not associated with any of these deleterious effects (92).

Although there is evidence of benefit to AP in Fontan patients, pacemaker placement itself can be technically complex in this population. In ECC Fontan patients, where the conduit completely bypasses atrial tissue, lead placement requires a thoracotomy or other innovative approaches to reach the atria (101). Therefore, endocardial pacing (with transvenous leads to the atrium) often can only be accomplished in patients whom atrial tissue is present inside the Fontan circuit—the APC or ILT Fontan. Endocardial leads also promote thrombus formation around the lead (which sits inside the sluggish and passive venous blood flow of the Fontan circulation) with embolization to the lungs or the systemic circulation being a possibility (102, 103). Given the more significant risk for morbidity and, potentially, mortality in this population, the decision to pace must be done with much thought and consideration.

### Heart block

In single ventricle patients, who tend to be even more heart rate dependent (due to lack of a subpulmonary ventricle to aid preload to the systemic ventricle and thus stroke volume), it follows that heart block would be particularly poorly tolerated. NPC-QIC single-ventricle database studies have supported this, with reports of 39% mortality by 12 months of age in those with surgical heart block—a more likely event compared to those single ventricle patients without heart block (OR 4.9, 95% GI 1.4–17.5, *p* = 0.01) (104). The resulting loss of atrioventricular synchrony is additionally problematic in Fontan patients, with studies showing association with increased hepatic pressures and clinical worsening (89). Chronic ventricular pacing has also been shown to be detrimental to single ventricle function over time. Bulic et al. evaluated 22 paced vs. 53 control (non-paced single ventricle) patients longitudinally in terms of clinical and echocardiographic parameter changes (100). The authors found that the paced patients were more likely to develop moderate to severe systolic dysfunction (68 vs. 15%, *p* < 0.01) and atrioventricular valve regurgitation (65 vs. 21%, *p* < 0.01) as well as require heart failure medications (65 vs. 21%, *p* < 0.01) or experience death or heart transplantation (odds ratio 4.9, 9% CI 1.05–22.7, *p* = 0.04) (100).

Histologic and pathologic causes for worsening ventricular function and outcomes in ventricularly paced patients have been described (105) and single ventricles with unique pressure and volume-loading challenges would seem at particular risk. To navigate this “rock and a hard place,” cardiac resynchronization therapy (CRT) has been proposed. Effectiveness, however, at least compared to in other patient populations, has not been extensively described. As an example, Dubin et al. reported that amongst 7 single-ventricle patients who underwent CRT/multisite resynchronization in their study, no significant improvement in systolic function was seen (106). Difficulties in understanding dyssynchrony in this patient population, as well as concrete issues such as exact location of lead placement, were postulated to be reasons for this lack of response (106). A larger review compared *n* = 19 CRT paced Fontan patients vs. *n* = 43 who were single-site ventricular paced (19). While there was no statistical difference in long-term outcomes, including ventricular systolic function and mortality, the authors acknowledged their study was likely underpowered to demonstrate the trend toward better outcomes in the CRT Fontans (19). Published guidelines including the 2021 PACES Expert Consensus Statement help with decision making in these complex patients (107). Pacing is a class I indication in patients with complex congenital heart disease with heart block resulting in hemodynamic compromise or a mean ventricular rate less than 60–70 bpm (107). This is a lower threshold than in patients without congenital heart disease, where a recommended mean ventricular rate <50 bpm is advised (107). The expert panel notes the additional functional and structural lesions, as well as more easily compromised hemodynamics, as reasons behind this difference.

### Monitoring and surveillance

There is little evidence to guide the frequency of monitoring for arrhythmias in Fontan patients. The 2019 American Heart Association guidelines recommend Holter monitoring every 2–3 years in children and every 1–2 years in adolescents and adults (1). However, the guidelines were not rooted in strong evidence and instead on the consensus of the authors (1). In a survey of centers with existing Fontan care programs, 27% obtain Holter monitoring annually while 36% of centers perform them every other year (108).

Saley et al. conducted a retrospective review of their institutional Fontan ambulatory rhythm surveillance program to study its utility (109). The protocol included routine use of ambulatory rhythm monitors at ages 6, 10, 13, 16, and 19 years of age (109). Eighty-three patients were included in the study with a total of 134 unique studies. Routine surveillance was the indication for 56% of the studies, with the remainder ordered for an abnormal electrocardiogram (28%), prior arrhythmias (36%), or reported symptoms (36%) (109). Indicated studies were more likely to find arrhythmia than surveillance studies (52% vs. 26%, *p* < 0.01) (109). Occult arrhythmias (not anticipated, found on surveillance studies) were responsible for 39% of positive ambulatory tests. The most common occult arrhythmia was SVT (10/23, 43%) with ventricular ectopy (9/23, 39%), accelerated junctional rhythm (3/23, 13%), and SND (1/23, 4%) also found (109). The most common arrhythmia on clinically indicated studies was accelerated junctional rhythm (15/45, 33%), with SVT (13/45, 29%), complex ventricular ectopy (11/45, 24%), Wenckebach rhythm (4/45, 9%), and SND (2/45, 4%) also being reported (109). Arrhythmia surveillance was increased in response to three (15%) studies with occult arrhythmias (1 SVT, 2 complex ventricular ectopy), and detection of any arrhythmia (occult or symptomatic) led to a change in clinical management in 31% of positive tests (14 patients with increased surveillance with the remaining two resulting in medications changes) (109).

Another study from Czosek et al. examined the utility of Holter monitoring in several different patient populations including those with Fontan physiology (110). Of the *n* = 51 Fontan patients undergoing a total of 148 Holter studies, 79% of the studies were obtained in asymptomatic patients (110). Of this Fontan cohort, the researchers found that only 9 of the 148 (6%) Holters lead to a change in clinical management (110). Of these, 5 of the patients underwent electrophysiology studies, four received pacemakers, and one had an implantable cardioverter-defibrillator device placed (110). The study examined the predictive values of Holter monitoring for detection of a significant arrhythmia event, including SVT, VT, SCD, and a history of appropriate ICD device therapy. The positive predictive value was poor (8%), however the negative predictive value was high (94%) (110). The study did not comment on a suggested frequency of monitoring. As such, following reported guidelines remains reasonable for now, with the hope that further studies will better inform these decisions over time.

### Conclusions

While the full scope of both the prevalence as well as the clinical implications of bradyarrhythmias in the Fontan population are still poorly defined, there is undoubtedly evidence that it is a common complication of the procedure and is likely underappreciated. Despite modifications to the Fontan procedure, arrythmias persist. It appears that the incidence of sinus node dysfunction increases as a function of time with many progressing to junctional rhythm. While pacing has been considered as a therapy for this patient population, the evidence to support this practice is inconclusive. Current guidelines recommend surveillance with Holter monitor every 2–3 years in adolescents and every 1–2 years in adults. Future directions for research include further assessment of junctional rhythm and its management as well as further identifying patients in which pacing would be beneficial.
